# Newly compiled Tai Chi (Bafa Wubu) promotes lower extremity exercise: a preliminary cross sectional study

**DOI:** 10.7717/peerj.15036

**Published:** 2023-03-13

**Authors:** Haojie Li, Fang Peng, Shaojun Lyu, Zhongqiu Ji, Xiongfeng Li, Mingyu Liu

**Affiliations:** 1 School of P.E and Sports, Beijing Normal University, Beijing, Haidian, China; 2 Department of PE, Peking University, Beijing, Haidian, China

**Keywords:** Tai Chi (Bafa Wubu), AnyBody musculoskeletal model, Muscle, Joint, Sport health

## Abstract

**Background:**

Tai Chi (Bafa Wubu) is a new type of simplified Tai Chi widely practiced by Tai Chi enthusiasts that has developed and perfected simplified Tai Chi movement and enriched Tai Chi practice methods. When practicing, Tai Chi athletes and enthusiasts can choose the Bafa Wubu movements to practice according to their physical conditions. The purpose of this article is to discuss the mechanism by which Bafa Wubu promotes lower extremity exercise from the perspective of exercise biomechanics.

**Objectives:**

This article aims to explore the scientific training methods and technical characteristics of Bafa Wubu, and its contribution to comprehensive exercise of the lower extremities, by analyzing the biomechanical characteristics of the lower extremities of participants who practice Bafa Wubu at different levels and by comparing their ground reaction force, lower limb joints, and muscles during Bafa Wubu.

**Methods:**

A total of 16 male participants were recruited and divided into an amateur group (*N* = 8) and a professional group (*N* = 8). The data were collected by a BTS 3D infrared-based motion capture system, and Kistler 3D force plate. The lower extremity joint forces and muscle strength were calculated by anybody simulation software with inverse dynamics.

**Results:**

During elbowing and leaning sideways with steps sideways (ELS), the ground reaction force of the professional group was significantly higher than that of the amateur group in the sagittal, vertical, and frontal axes (*P* < 0.01). While stepping forward, backward, and sideways, the professional group’s joints loading at the hip, knee, and ankle was always higher in the vertical direction (*P* < 0.01). Furthermore, during warding off with steps forward (WOF), laying with steps forward (LF), and rolling back with steps backward (RBB), hip joint loading increased in the med–lat direction. During actions with steps backward and sideways, the professional group’s ankle flexion/extension torque and hip abduction/rotation torque were significantly larger than those of the amateur group (*P* < 0.01). Different actions in Bafa Wubu activate muscles to different degrees, whereas the iliacus is mainly responsible for stabilizing postures when practitioners perform standing knee lifting motions.

**Conclusions:**

Professional groups who have been practicing Tai Chi (Bafa Wubu) for a long time have higher ground reaction force, and the force on the three joints of the lower extremities is different for various movements, which has positive significance for exercising the joints of the lower extremities. In addition, various motions activate muscles of different types at different levels. For amateurs to practice different movements to stimulate the muscles, targeted areas of practice promote the lower extremity muscles’ synergistic force. In summary, the muscles and joints of the lower extremity can obtain comprehensive and balanced exercise through Bafa Wubu.

## Introduction

Decreased lower extremity mobility affects people’s quality of life (Uysal et al., 2022), especially in the elderly, and is accompanied by loss of muscle strength and decreased control of the joints (Evans, 1995), which is often accompanied by many motor risks (Hortobagyi & DeVita, 1999), such as: decreased muscle strength increases the burden on joints and muscles and accelerates cartilage degeneration (Lai et al., 2021). As a result, older adults need to use more muscles and strength to support the body and perform more activities (Haggerty et al., 2014). In addition, decreased lower extremity mobility is more likely to result in lower extremity sports injuries (Mora, 2018), which can often cause many inconvenient experiences if they occur. It is especially obvious when performing weight-bearing activities, running training or walking (Kammerlander et al., 2012). Therefore, it is very meaningful to improve the motor ability of the lower limbs through exercise. Tai Chi has become a widespread exercise worldwide as a complementary and alternative therapy for the treatment of lower extremity injuries (Kallinen & Markku, 1995). The health-promoting effects of Tai Chi have been extensively studied in past research, (Esposito et al., 2017) who showed that Tai Chi is a safe and effective way to promote physical health (Pan et al., 2015). It can effectively slow down the loss of muscle strength and enhance the control of lower limb joints (Lee, Lee & Ernst, 2009; Koren, Leveille & You, 2021). Currently, Tai Chi has been widely used in clinical practice.

As an ancient Chinese martial art, Tai Chi’s role in fitness and health has interested many scholars. Past studies have shown that practicing Tai Chi can strengthen the lower extremities (Ge et al., 2022), reduce bone density loss (Zou et al., 2017), improve balance (Wang et al., 2021), and prevent falls (Li et al., 2021). In a related study, Ghandali et al. (2017) further supported the effectiveness of Tai Chi in improving ankle motion by comparing the effects of normal walking and Tai Chi intervention on proprioception, showing that the ankle flexor muscle groups were significantly active and enhanced ankle proprioception during Tai Chi training. The study of Jagodinsky et al. (2015) confirmed that standardized Tai Chi movements could avoid medial knee loading and reduce the risk of a knee injury. Furthermore, future research needs to model the standardization of Tai Chi. It will help amateurs practice Tai Chi in a more standardized manner. In addition, Kuo et al. (2021) showed that Tai Chi training could significantly increase the gap between the foot and the obstacle and can effectively reduce the risk of falls in the elderly. Tai Chi also has a favorable effect on muscle activation training. Li & Law (2018) showed that the slow movements and higher joint loads of Tai Chi allowed more muscle groups of the lower extremity muscles to participate in active movement. Moreover, Tai Chi can increase the range of anterior and posterior knee motion by using body weight displacement, enhancing neuromuscular exercise of the lower limb. Wu & Ren (2009) studied leg muscle activity during Tai Chi exercise and found that the speed of Tai Chi exercise influenced the activation time and function of knee extensor muscles. The speed of Tai Chi exercise changes the form and role of muscle contraction during exercise. Through clinical studies, Zhang et al. (2015) found that Tai Chi limb movements were larger, joint loads grew more slowly, and muscle activity was higher. Therefore, Tai Chi exercise is helpful for enhancing the muscle strength and function of the legs in patients with osteoarthritis. According to current research, there is some evidence that Tai Chi positively effects on physical function.

Tai Chi (Bafa Wubu) studied in this article is a new type of simplified Tai Chi that is suitable for practice by the general population, such as older people whose cognitive ability decreases with age and their ability to perform and learn (Corbo & Casagrande, 2022; Ngandu et al., 2015). Older people have difficulty completing complex tasks (Fezzani et al., 2010), while traditional Tai Chi training methods are more complex for older people. It is difficult to practice (Lei et al., 2022). Considering that Tai Chi of the traditional form is difficult for elderly individuals, simplified and personalized versions have been developed for certain groups (Chen et al., 2020). Hence, the General Administration of Sport of China promoted a more user-friendly routine of Tai Chi based on the 24-form simplified Tai Chi, called Bafa Wubu (Lyu et al., 2020). It integrates eight essential forms called Peng (warding off), Lv (rolling back), Ji (pressing), An (pushing), Cai (plucking), Lie (laying), Zhou (elbowing), Kao (leaning sideways), and five steps called Jin (advancing), Tui (retreating), Gu (shifting left), Pan (shifting right), and Ding (central equilibrium), which are the core patterns of Tai Chi. Compared with the 24-form simplified version, Bafa Wubu is simpler and takes less time and energy (Lyu et al., 2020). Therefore, it is of great value to probe Bafa Wubu’s role and mechanism of promoting fitness.

Chen et al. (2012) found that by practicing Tai Chi (Bafa Wubu), elderly individuals enhance their proprioception, promote their vestibular function and strengthen their lower extremities, subsequently improving their dynamic and static balance. Likewise, Hu, Song & Zhang (2021) reported that Bafa Wubu improves the foot center of pressure (COP) and foot arch tactile sense more than knee proprioception so that the elderly has enhanced balance capacity and a reduced risk of falls. According to the literature, Bafa Wubu of Tai Chi was launched relatively recently, and its biomechanical research is limited. There is less exploration of its movement mechanism; therefore, the innovation of this thesis is to use computer simulation modeling to analyze the movement biomechanical characteristics of the lower limb of Tai Chi (Bafa Wubu) exercise, thus supplementing the research gap in this area. Moreover, most of the previous Tai Chi studies have been analyzed for single movements, while this is a comprehensive study with the complete movements of Bafa Wubu.

The research hypothesis of this article proposes that (1) ground reaction forces will increase in practitioners who train Bafa Wubu for more than 5 years. (2) Increased joint forces and joint moments in the lower extremities of practitioners who train Tai Chi (Bafa Wubu) for more than 5 years. (3) Practitioners who train Tai Chi (Bafa Wubu) for more than 5 years will have increased muscle strength in the lower extremities.

## Materials and Methods

### Participants

Prior to experimental testing, we received informed consent from all participants.

The present experiment is a preliminary study based on a small group of subjects. A total of 16 male volunteers were recruited as subjects. Half served as the professional group, while the other half served as the amateur group. Only professional athletes who had achieved both national level titles and placed in the top three in domestic Tai Chi competitions were qualified for the professional group, and practicing Bafa Wubu for more than 5 years, Male teenagers practicing Bafa Wubu for more than 1 year were qualified for the amateur group. Then, five national master’s sportsmen and three athletes at the national level were recruited from Beijing Sport University, making up the professional group. The amateur group was recruited from students attending Beijing Normal University. All subjects were in good health with no history of lower extremity injury in the past 6 months, and everyone’s dominant leg was on the right side. There were no significant differences in physical dimensions between the two groups based on the subjects’ records, including age, height, weight, BMI, leg length, hip width, knee width, or ankle width. All participants provided written informed consent and understood the experimental process and purpose. All methods in this study were carried out following the ‘Declaration of Helsinki’ relevant guidelines and regulations, and this study was reviewed and approved by the Ethics Committee of China National Rehabilitation Center (No. S20220206). The participants’ basic information is listed in Table 1.

**Table 1:** Basic information of all participants (mean ± SD).

Body parameters	Groups		T value	*P* value
	Amateur group (*N* = 8)	Professional group (*N* = 8)		
Age (y)	21.50 ± 2.14	20.50 ± 1.60	1.058	0.31
Height (cm)	174.44 ± 4.95	175.00 ± 5.24	−0.221	0.83
Weight (kg)	70.25 ± 6.20	70.75 ± 3.77	−0.195	0.85
BMI	23.10 ± 2.05	23.13 ± 1.49	−0.038	0.97
Leg length (cm)	86.25 ± 0.71	85.94 ± 2.40	0.354	0.73
Hip width (cm)	22.50 ± 1.85	23.13 ± 1.96	−0.656	0.52
Knee width (cm)	9.50 ± 0.65	9.94 ± 0.68	−1.313	0.21
Ankle width (cm)	7.25 ± 0.60	7.38 ± 0.52	−0.447	0.66

**Note:**

*P* > 0.05 represents insignificant differences among the basic information of the two groups. Due to the difference of the skeletal muscle model, the model parameters need to be changed according to subjects’ body shape, hence body shape parameters of the subjects are collected.

### Instrumentation

In this study, all motion data were collected by eight high-precision infrared motion capture systems (BTS SMART DX 700, BTS Bioengineering, Garbagnate Milanese, Italy), whose parameters are as follows: frequency of 250 Hz, resolution of 640 dpi × 480 dpi pixels, the precision of 400 mm × 300 mm × 300 mm. Three 3D force plates (928E, Kistler, Winterthur, Switzerland) were used to measure the ground reaction force with a frequency of 1,000 Hz and a static detection error of less than 0.5%.

### Tai Chi (Bafa Wubu)

**Figure 1:**
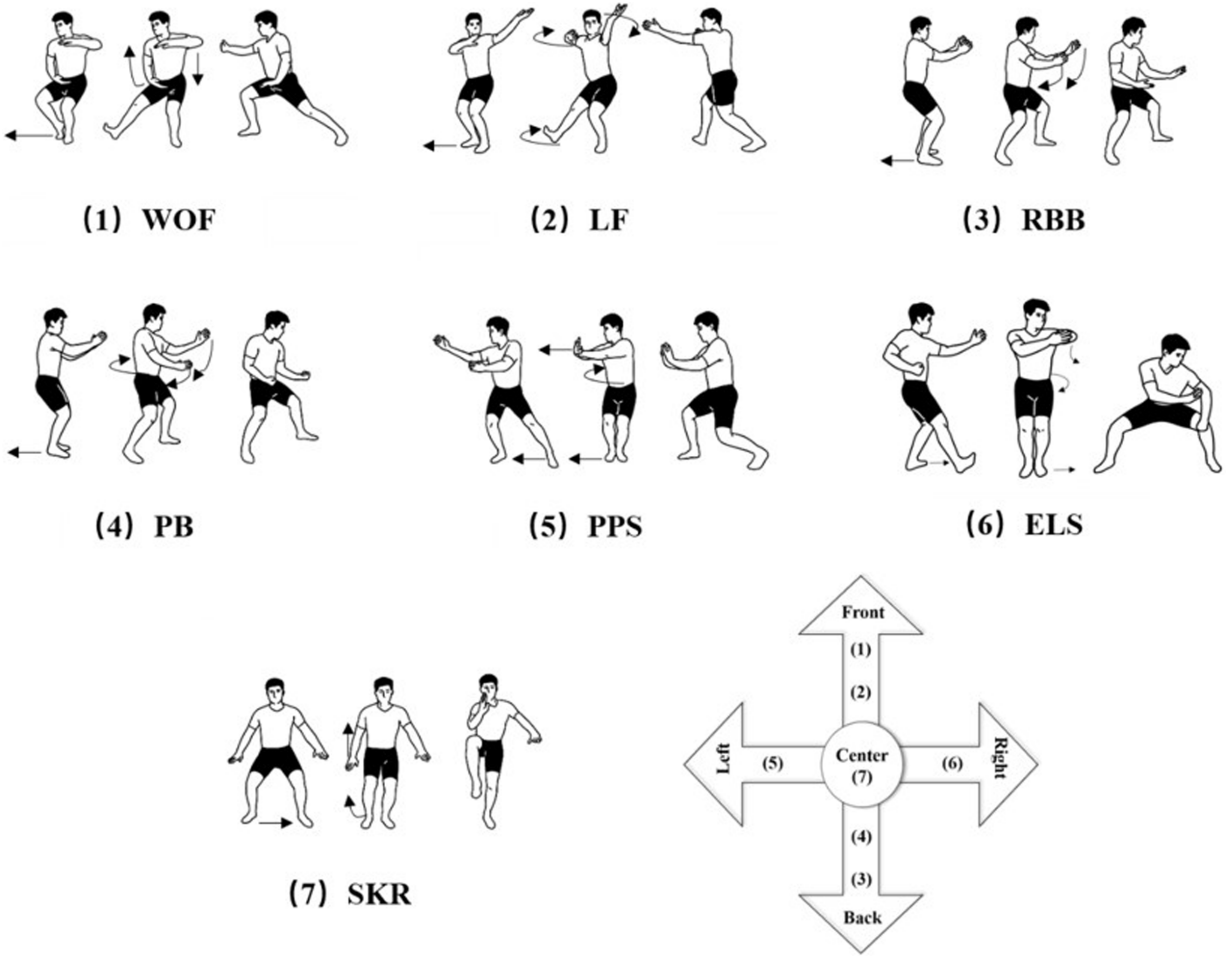
Illustration of the seven sets of movements of the Taijiquan Bafa Wubu. (1) WOF: step forward, (2) LF: step forward, (3) RBB: roll backward, (4) PB: backward swing, (5) PPS: side push and press, (6) ELS: elbow and lean, lateral swing, (7) SKR: standing knee raise.

Bafa Wubu, a primary routine of Tai Chi, includes five types of footwork called Jin, Tui, Gu, Pan, and Ding, which, respectively, mean moving forward, backward, left, right, and standing still. As shown in Fig. 1, by combining the five types of footwork and eight essentials, Bafa Wubu can be divided into seven sets of motions: WOF (warding off with steps forward), LF (laying with steps forward), RBB (rolling back with steps backward), PB (plucking with steps backward), PPS (pushing and pressing with steps sideways), ELS (elbowing and leaning sideways with steps sideways), and SKR (standing knee raises). Namely, WOF and LF move forward, RBB and PB move backward, PPS and ELS move sideways, and SKR is a balanced and motionless posture. These motions include the 13 fundamental techniques at the core of Tai Chi with different features in the directions of displacement, strength generation methods and exercise effects; therefore, the seven sets of motions above were selected as the subject of this research.

### Simulation system

A musculoskeletal simulation model is established through anybody 7.2 software (AnyBody Technology, Aalborg, Denmark) to process 3D motion-capture dynamics. Validated by many experiments, Anybody 7.2 software runs with high reliability and precision (Saraswat, Andersen & Macwilliams, 2010; Damsgaard et al., 2006). The musculoskeletal model established in AnyBody is a standard multibody dynamic model that consists of rigid parts (such as the human skeleton or external objects), kinematic actuators (such as body motion), and force/torque actuators (such as muscles). Generally, forces and torques are simulated using multibody dynamics simulation.

### Testing protocols

This experiment was completed in the sports biomechanics laboratory of Beijing Normal University. We set BTS infrared motion capture cameras at intervals of more than 200 mm and at the height of 300 mm. The cameras were placed in a semiarc around the test center. Before the experiment, the global coordinates and force plate coordinates were calibrated to ensure that each camera could capture the participants’ motions and bodies. A VIXTA recording camera was used to record the whole experiment.

All subjects were acquired on the same day, and data were acquired sequentially, with three data acquisitions per action and 2,000 frames. After all data were collected, the next person’s data were collected 15 min later.

**Figure 2:**
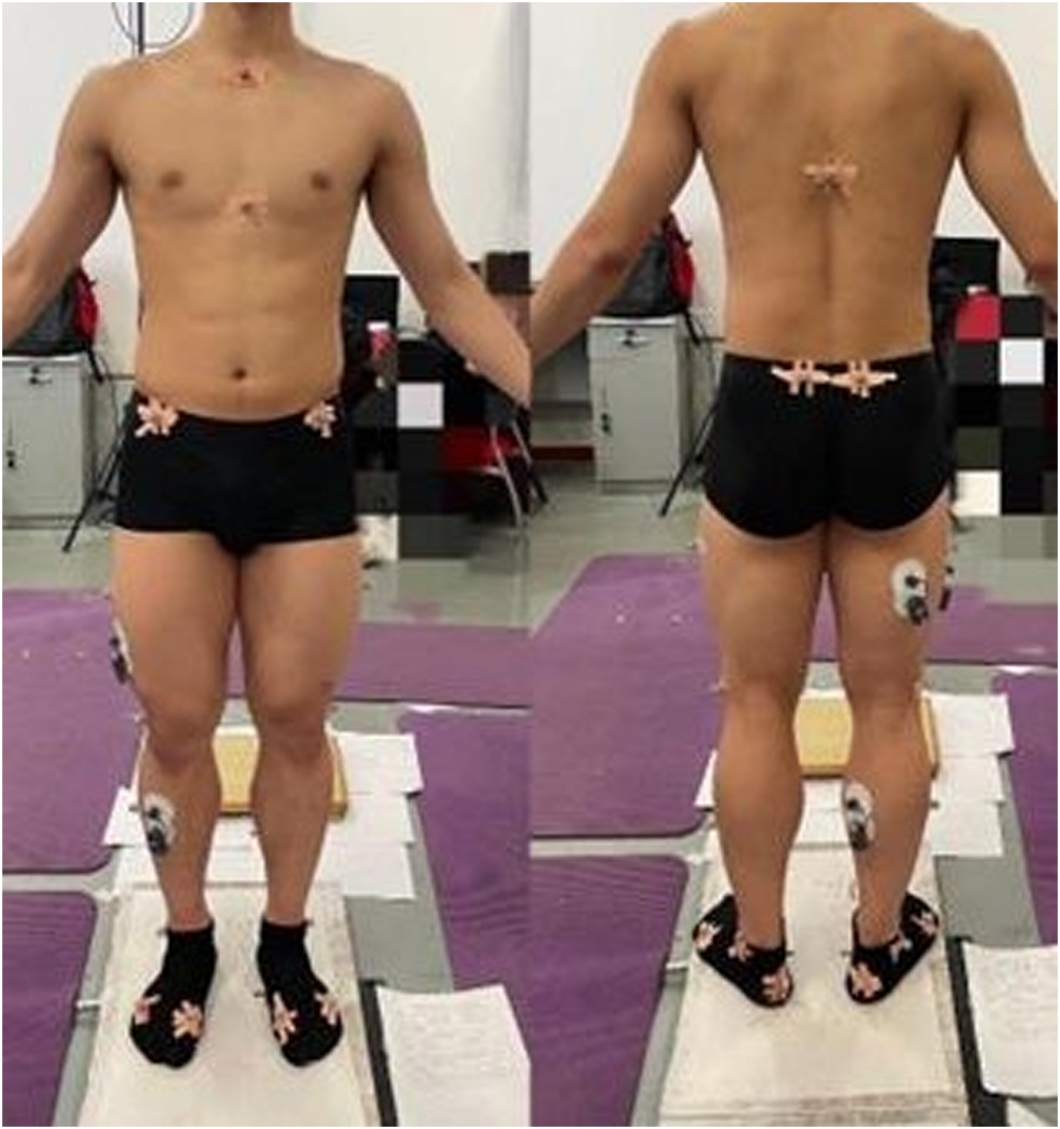
A total of 25 marker points were attached to the bone marker points of the subject.

Participants wore uniform shorts and black socks to reduce errors due to clothing shaking and other variables. According to the requirements of the plugin Gait, the marker set Lower Extremity model in AnyBody 7.2, 25 marker points were attached to the bone marker points of the subjects. The positions of marker points included the left and right anterior head, left and right posterior head, sternum, clavicle joint, tenth thoracic vertebra, sternal xiphoid process, left and right anterior superior iliac spine, left and right posterior superior iliac spine, left and right lateral lower 1/3 of the thigh, left and right external epicondyle of the fibula, right lateral lower 1/3 of the calf, left lateral lower 1/2 of the leg, left and right heel, left and right lateral malleolus, left and right first metatarsal, and left and right fifth metatarsal (Fig. 2) (Schwarze et al., 2013; Manders, New & Rasmussen, 2008).

### Data collection and analysis

Since all participants were right-side dominant and the motions are symmetric and periodic in Bafa Wubu of Tai Chi, this experiment mainly investigated the characteristics of motions when the force of the knee joint in the vertical direction reaches the maximum in the stance phase of the right lower extremity, which lasts from heel strike to toe-off (Babaee, Li & Rigoll, 2018).

### Force plate data processing

The force plate and electromyography data were processed using a BTS SMART Analyzer. We determined the range of the force plate according to the data and determined its maximum values from periods processing of force in the X, Y and Z axes (Liu et al., 2008).

### Dynamic data of the lower extremities

**Figure 3:**
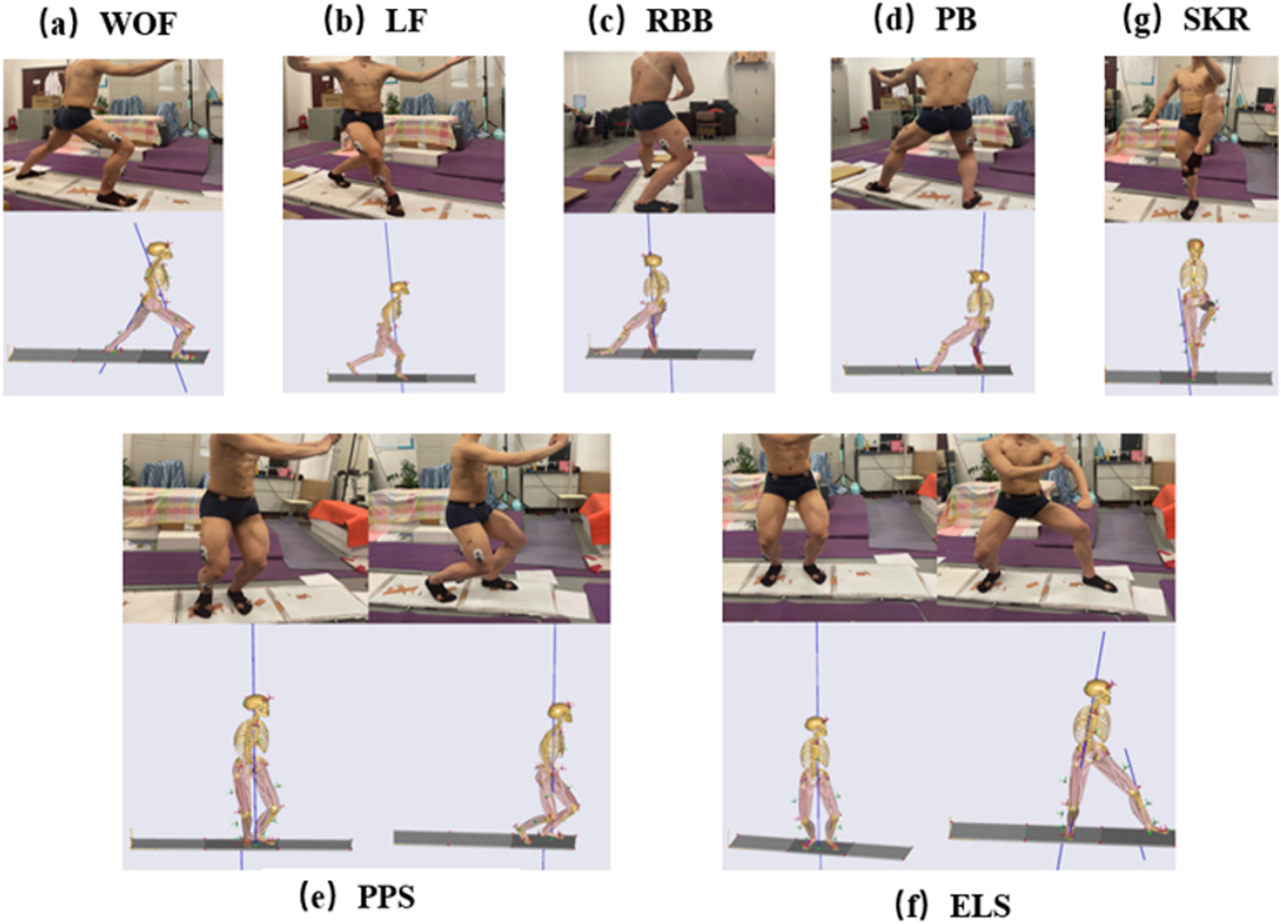
Musculoskeletal models of seven sets of motions in Bafa Wubu of Tai Chi. (A) WOF, (B) LF, (C) RBB, (D) PB, (E) PPS, (F) ELS, and (G) SKR.

We used BTS SMART Capture software and eight BTS SMART Dx 700 cameras to shoot the motion data of the reflective markers and then delineated their moving paths by the BTS tracker. When the process was finished, the C3D file of kinematic data was imported into AnyBody 7.2 simulation software to establish a musculoskeletal simulation model of the seven sets of Tai Chi motions (Fig. 3).

The calculation process of the body simulation is as follows: optimizing the reflective markers, conducting the dynamics computation, and conducting inverse dynamics computation. Then, the dynamic data were calculated. Standardized processing was accomplished after the calculated dynamic indices were imported into Excel. In addition, joint force, joint torque, and muscle strength were divided by body weight (unit: N/BW) (Walter & Pandy, 2017), where joint forces were defined to be both positive and negative values between the joints (Wu et al., 2005).

### Statistical analysis

This study, analyzed data with statistical software (SPSS 26.0), and the results are displayed as the mean value and standard deviation (mean ± SD). Independent samples t-test were used to analyze the dynamic data of the lower extremities of Bafa Wubu practitioners at different levels.

## Results

### Comparison of the ground reaction forces

**Figure 4:**
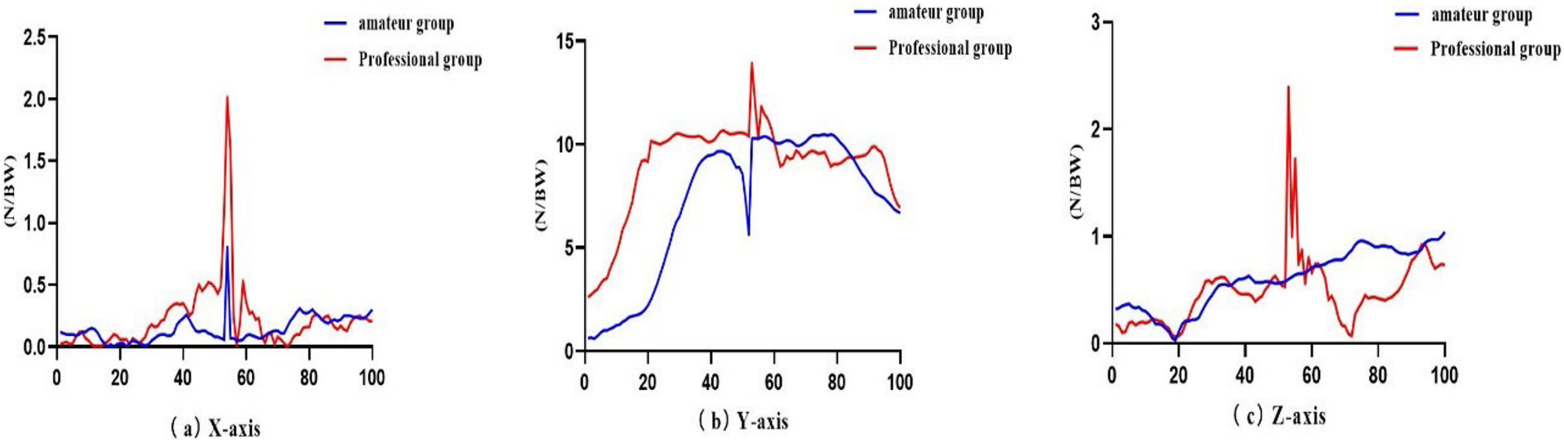
Comparisons of the GRF of the amateur group (blue line) and the professional group (red line) during ELS. (A) GRF on the X-axis, (B) GRF on the Y-axis, and (C) GRF on the Z-axis.

As shown in Table 2 and Fig. 4, during ELS, the GRF of the professional group was significantly higher than that of the amateur group in the vertical, anterior–posterior (ant–post), and medial–lateral (med–lat) directions (*P* < 0.01).

**Table 2:** Mean and standard deviation (mean ± SD) of the maximum ground reaction force (GRF) during seven sets of motions. Units: N/BW.

	Groups	WOF	LF	RBB	PB	PPS	ELS	SKR
F(x)	Amateur	0.43 ± 0.08	0.38 ± 0.11	0.50 ± 0.15	0.61 ± 0.23	0.99 ± 0.83	0.80 ± 0.85	0.26 ± 0.07
	Professional	0.51 ± 0.10	0.42 ± 0.12	0.45 ± 0.06	0.51 ± 0.11	1.71 ± 1.04	2.03 ± 1.00**	0.36 ± 0.21
F(y)	Amateur	9.47 ± 2.53	10.03 ± 1.13	10.89 ± 0.83	10.73 ± 1.02	12.23 ± 1.53	10.71 ± 2.10	5.56 ± 0.45
	Professional	9.73 ± 0.77	10.13 ± 0.37	10.20 ± 0.28	10.12 ± 0.56	11.29 ± 1.18	13.05 ± 1.66**	5.74 ± 1.84
F(z)	Amateur	1.20 ± 0.28	1.15 ± 0.33	1.11 ± 0.42	1.29 ± 0.57	1.45 ± 0.40	1.39 ± 0.32	0.89 ± 0.15
	Professional	1.42 ± 0.21	1.15 ± 0.30	1.03 ± 0.25	0.92 ± 0.26	1.95 ± 0.67	2.22 ± 0.67**	0.96 ± 0.94

**Note:**

** *P* < 0.01 represent the comparison between the professional group and the amateur group; the X-axis is the sagittal axis, the Y-axis is the vertical axis, and the Z-axis is the frontal axis.

### Comparisons of joint load of the lower extremity

The joint reaction forces exerted on the ankle, knee, and hip joints are shown with peak values in Table 3, where the X, Y, and Z axes represent the med–lat, vertical, and ant–post directions, respectively.

**Table 3:** Mean and standard deviation (mean ± SD) of the maximum joint force of the hip, knee and ankle during the seven sets of motions. Units: N/BW.

Joint force		Group	WOF	LF	RBB	PB	PPS	ELS	SKR
**Hip**	**X-axis**	Amateur	−0.77 ± 0.48	−1.02 ± 0.70	−0.62 ± 0.34	−0.57 ± 0.26	−0.73 ± 0.27	−1.22 ± 0.98	4.96 ± 3.46
		Professional	−0.12 ± 0.09	−0.75 ± 0.46	−0.47 ± 0.28	−0.75 ± 0.98	−0.67 ± 0.42	−0.55 ± 0.57	10.69 ± 5.51**
	**Y-axis**	Amateur	30.69 ± 16.19	29.95 ± 13.20	30.06 ± 15.55	57.49 ± 24.09	87.98 ± 53.09	51.98 ± 23.40	14.16 ± 8.60
		Professional	74.62 ± 39.66**	76.95 ± 41.47**	117.53 ± 99.00**	75.37 ± 26.89	101.67 ± 63.09	70.97 ± 27.00	23.95 ± 12.77
	**Z-axis**	Amateur	4.10 ± 3.90	3.56 ± 3.43	2.41 ± 1.83	13.50 ± 9.17	23.48 ± 20.15	9.35 ± 5.52	1.93 ± 0.95
		Professional	20.75 ± 14.30**	21.28 ± 6.39**	27.41 ± 12.58**	27.00 ± 14.12	32.86 ± 19.21	12.99 ± 3.87	2.85 ± 1.47
**Knee**	**X-axis**	Amateur	−2.18 ± 3.63	−1.73 ± 0.85	−0.82 ± 0.42	−0.90 ± 0.41	−0.46 ± 0.21	−3.96 ± 1.18	−1.35 ± 0.64
		Professional	−0.56 ± 2.37	−1.15 ± 0.32	1.80 ± 1.14	−1.03 ± 1.16	0.05 ± 0.02	0.46 ± 0.13	−3.77 ± 1.79
	**Y-axis**	Amateur	2.83 ± 1.46	2.39 ± 1.39	4.70 ± 3.03	3.85 ± 2.24	7.43 ± 3.91	6.56 ± 3.54	0.81 ± 0.17
		Professional	3.73 ± 3.11	7.32 ± 2.95 **	10.85 ± 4.80**	8.07 ± 3.92 **	11.58 ± 1.91**	11.24 ± 3.92**	1.69 ± 1.15
	**Z-axis**	Amateur	−0.99 ± 1.05	−0.49 ± 0.85	−0.02 ± 0.01	−0.04 ± 0.03	0.10 ± 0.01	−5.57 ± 4.53	−0.37 ± 0.43
		Professional	0.32 ± 0.43	0.15 ± 0.11	−0.05 ± 0.03	−0.26 ± 0.14	0.11 ± 0.07	0.02 ± 0.02	−1.59 ± 1.21
**Ankle**	**X-axis**	Amateur	1.26 ± 1.06	1.76 ± 1.52	4.25 ± 1.83	4.58 ± 1.88	3.72 ± 1.29	3.01 ± 1.79	6.95 ± 2.67
		Professional	3.13 ± 2.86	3.54 ± 2.90	4.74 ± 1.67	3.91 ± 2.55	4.35 ± 1.24	5.40 ± 3.49	6.52 ± 0.95
	**Y-axis**	Amateur	13.65 ± 5.81	13.42 ± 4.37	19.11 ± 10.25	18.62 ± 7.78	90.45 ± 28.76	60.94 ± 22.12	21.48 ± 17.27
		Professional	18.29 ± 8.06	18.37 ± 13.23	34.77 ± 15.92**	23.07 ± 13.20**	148.97 ± 30.94**	87.42 ± 33.65	10.65 ± 7.90
	**Z-axis**	Amateur	−1.04 ± 1.44	−1.31 ± 1.09	−0.08 ± 0.11	−0.15 ± 0.10	−0.08 ± 0.10	−8.87 ± 2.05	−0.05 ± 0.10
		Professional	−0.11 ± 0.20	−0.20 ± 0.21	−0.05 ± 0.09	−0.37 ± 0.74	−0.07 ± 0.18	−6.17 ± 2.14	−2.39 ± 6.92

**Note:**

** *P* < 0.01 represents comparisons between the amateur group and the professional group. The X-axis is the sagittal axis, the Y-axis is the vertical axis and the Z-axis is the frontal axis.

In terms of hip joints, during SKR, the hip joint reaction force of the professional group in the med–lat direction was higher than that of the amateur group (*P* < 0.05); during WOF, LF, and RBB, the hip joint reaction forces of the professional group were significantly higher than those of the amateur group in both the vertical and ant–post directions (*P* < 0.01).

In terms of knee joints, during LF, RBB, PB, PPS, and ELS, the knee joint reaction force of the professional group was significantly higher than that of the amateur group in the vertical direction (*P* < 0.01). In contrast, no significant differences existed in the med–lat and ant–post directions.

In terms of ankle joints, during RBB, PB, and PPS, the ankle joint reaction forces of the professional group were significantly higher than those of the amateur group in the vertical direction (*P* < 0.01). However, no significant differences existed in the med–lat and ant–post directions.

As shown in Table 4, during RBB, hip abduction torque, hip flexion/extension torque and knee flexion/extension torque of the professional group were significantly larger than those of the amateur group (*P* < 0.01), while the hip rotation torque of the professional group was larger, but not significantly, than that of the amateur group (*P* < 0.05).

**Table 4:** Mean and standard deviation (mean ± SD) of the maximum joint torque of the hip, knee and ankle during the seven sets of motions. Units: Nm/BW.

Joint torque	Group	WOF	LF	RBB	PB	PPS	ELS	SKR
Hip abduction	Amateur	0.28 ± 0.10	0.28 ± 0.22	0.61 ± 0.23	0.55 ± 0.26	1.00 ± 0.23	0.89 ± 0.36	0.27 ± 0.12
	Professional	0.41 ± 0.13	0.53 ± 0.27	1.26 ± 0.76**	0.90 ± 0.26**	1.25 ± 0.71	1.04 ± 0.21	0.21 ± 0.10
Hip rotation	Amateur	0.19 ± 0.08	0.21 ± 0.14	0.10 ± 0.09	0.11 ± 0.09	0.32 ± 0.18	0.25 ± 0.11	0.09 ± 0.04
	Professional	0.34 ± 0.24	0.20 ± 0.07	0.35 ± 0.19**	0.08 ± 0.04	0.51 ± 0.23	0.42 ± 0.21	0.08 ± 0.04
Hip flex/ext	Amateur	0.40 ± 0.17	0.39 ± 0.37	0.30 ± 0.17	0.32 ± 0.24	0.64 ± 0.33	0.09 ± 0.07	0.38 ± 0.13
	Professional	0.38 ± 0.14	0.36 ± 0.23	1.37 ± 1.06**	0.59 ± 0.25**	0.81 ± 0.42	0.88 ± 0.83**	0.37 ± 0.04
Knee flex/ext	Amateur	0.39 ± 0.24	0.28 ± 0.20	0.10 ± 0.01	0.09 ± 0.01	0.20 ± 0.11	0.36 ± 0.29	0.29 ± 0.19
	Professional	0.53 ± 0.46	0.33 ± 0.26	0.54 ± 0.08**	0.18 ± 0.12	0.22 ± 0.10	0.44 ± 0.45**	0.34 ± 0.26
Ankle flex/ext	Amateur	0.32 ± 0.28	0.44 ± 0.36	0.90 ± 0.38	0.57 ± 0.21	0.94 ± 0.24	0.70 ± 0.29	0.66 ± 0.14
	Professional	0.55 ± 0.34	0.57 ± 0.45	0.91 ± 0.23	0.76 ± 0.09**	0.86 ± 0.21	0.92 ± 0.38	0.51 ± 0.36

**Note:**

** *P* < 0.01 represent comparisons of the amateur group and the professional group.

During PB, the professional group’s hip abduction torque, hip flexion/extension torque, and ankle flexion/extension torque were significantly larger than those of the amateur group (*P* < 0.01).

During ELS, the professional group’s hip flexion/extension torque and knee flexion/extension torque were significantly larger than those of the amateur group (*P* < 0.01).

During the remaining four sets of motions, there were no significant differences between the two groups’ net joint torque of the three joints.

### Comparisons of lower extremity muscle strength

Table 5 compares the lower extremity muscle strength between the two groups during seven motions.

**Table 5:** Mean and standard deviation (mean ± SD) of muscle strength during the seven sets of motions. Units: N/BW.

Muscles	Group	WOF	LF	RBB	PB	PPS	ELS	SKR
Rectus femoris	Amateur	3.28 ± 1.31	2.77 ± 1.47	8.27 ± 3.59	7.05 ± 2.10	8.47 ± 2.64	8.23 ± 2.60	1.70 ± 1.43
	Professional	3.95 ± 2.14	5.85 ± 3.50**	11.86 ± 3.07**	8.55 ± 3.05	13.10 ± 2.80**	10.71 ± 3.66	3.84 ± 2.86
Vastus lateralis	Amateur	10.86 ± 4.70	10.19 ± 5.50	25.50 ± 11.30	24.53 ± 8.18	28.82 ± 10.56	30.57 ± 12.25	6.72 ± 4.95
	Professional	11.70 ± 9.51	23.73 ± 10.35**	36.25 ± 12.75	29.58 ± 14.26	44.81 ± 6.48**	38.88 ± 16.06	2.73 ± 2.57
Vastus medialis	Amateur	5.63 ± 2.74	5.04 ± 2.73	13.15 ± 6.81	12.92 ± 2.86	15.18 ± 5.33	16.10 ± 6.32	3.30 ± 2.42
	Professional	7.10 ± 5.37	12.09 ± 4.59**	22.16 ± 7.14**	15.86 ± 8.69	24.97 ± 4.30**	22.15 ± 8.40	1.35 ± 1.27
Biceps femoris	Amateur	3.12 ± 2.28	4.72 ± 2.95	2.10 ± 0.79	3.66 ± 2.90	5.38 ± 3.46	1.74 ± 0.73	4.24 ± 2.50
	Professional	5.12 ± 2.29	5.11 ± 2.96	10.92 ± 10.17**	4.83 ± 2.66	8.29 ± 5.02	3.92 ± 1.18**	4.19 ± 2.70
Iliacus	Amateur	2.65 ± 1.58	2.17 ± 1.05	2.29 ± 1.36	1.59 ± 0.99	3.75 ± 4.26	1.57 ± 1.45	0.95 ± 0.86
	Professional	2.43 ± 1.19	2.15 ± 0.66	7.61 ± 6.09**	3.15 ± 0.88**	7.76 ± 2.98**	5.69 ± 2.19**	2.64 ± 1.67**
Gluteus maximus	Amateur	7.99 ± 4.74	8.20 ± 4.45	3.72 ± 2.23	8.76 ± 7.09	15.75 ± 8.13	13.46 ± 4.58	3.82 ± 2.21
	Professional	11.50 ± 5.61	13.47 ± 7.32	18.65 ± 15.01**	11.53 ± 18.03	18.89 ± 8.35	15.96 ± 6.63	1.85 ± 1.43
Gluteus minimus	Amateur	1.77 ± 1.03	1.87 ± 1.77	3.73 ± 1.99	3.30 ± 1.06	5.88 ± 1.88	5.21 ± 1.86	0.86 ± 0.57
	Professional	2.89 ± 1.06	3.84 ± 1.87**	8.29 ± 3.41**	3.93 ± 2.91	9.21 ± 3.83**	6.96 ± 2.93	1.02 ± 0.80
Sartorius	Amateur	1.83 ± 0.91	1.55 ± 1.28	1.38 ± 0.41	1.35 ± 0.74	2.14 ± 1.74	1.00 ± 0.87	1.30 ± 0.69
	Professional	1.85 ± 0.51	2.04 ± 0.51	2.54 ± 0.55	5.00 ± 1.24**	3.43 ± 2.59	3.56 ± 1.59**	1.69 ± 0.79
Adductor longus	Amateur	0.68 ± 0.76	0.95 ± 0.70	0.70 ± 0.39	0.66 ± 0.44	2.00 ± 1.45	0.71 ± 0.44	0.47 ± 0.31
	Professional	1.11 ± 0.88	0.40 ± 0.26	3.57 ± 2.82**	1.24 ± 0.71	2.67 ± 0.95	3.29 ± 2.24**	0.51 ± 0.45
Tibialis anterior	Amateur	0.81 ± 0.53	1.94 ± 1.64	0.16 ± 0.10	0.21 ± 0.13	5.42 ± 4.12	5.45 ± 4.73	1.33 ± 0.86
	Professional	4.87 ± 4.30**	4.85 ± 2.71**	0.80 ± 1.32	0.39 ± 0.27	4.85 ± 1.32	7.63 ± 2.10	3.04 ± 1.82
Tibialis posterior	Amateur	0.05 ± 0.01	0.62 ± 0.49	1.43 ± 0.67	0.22 ± 0.41	1.30 ± 0.90	1.72 ± 1.35	1.11 ± 0.32
	Professional	0.50 ± 0.46**	0.89 ± 0.73	1.98 ± 1.11	0.54 ± 0.27	1.42 ± 1.32	2.34 ± 1.32	1.07 ± 0.92
Gastrocnemius	Amateur	1.86 ± 0.31	3.49 ± 2.53	19.35 ± 10.79	16.27 ± 7.26	14.03 ± 5.00	10.82 ± 6.84	8.47 ± 2.87
	Professional	12.25 ± 3.38**	11.32 ± 3.10**	23.76 ± 13.65	19.64 ± 12.28	17.59 ± 9.06	14.38 ± 7.58	9.10 ± 8.08

**Note:**

** *P* < 0.01 represent comparisons of the amateur group and the professional group.

During WOF, the professional group showed significantly more strength than the amateur group for the tibialis anterior, tibialis posterior, and gastrocnemius (*P* < 0.01).

During LF, the professional group showed significantly more strength than the amateur group for the rectus femoris, vastus lateralis, vastus medialis, gluteus minimus, tibialis anterior, and gastrocnemius (*P* < 0.01).

During RBB, the professional group showed significantly more strength than the amateur group for the rectus femoris, vastus medialis, biceps femoris, iliacus, gluteus maximus, gluteus minimus, and adductor longus (*P* < 0.01).

During PB, the professional group showed significantly more strength than the amateur group for the iliacus and sartorius (*P* < 0.01).

During PPS, the professional group showed significantly more strength than the amateur group for the rectus femoris, vastus lateralis, vastus medialis, iliacus, and gluteus minimus (*P* < 0.01).

During ELS, the professional group showed significantly more strength than the amateur group for the biceps femoris, iliacus, adductor longus, and sartorius (*P* < 0.01).

During SKR, the professional group showed significantly more strength than the amateur group for the iliacus (*P* < 0.01).

## Discussion

### Ground reaction force activity analysis

Ground reaction force (GRF) is an essential metric in biomechanics. It can reflect the horizontal and vertical component forces generated by the lower limbs on the ground during different movements (Yu et al., 2021a). The study in this article found that among seven sets of motions, the GRF of the professional group was significantly higher than that of the amateur group in the vertical, anterior–posterior (ant–post), and medial–lateral (med–lat) directions. ELS is a lateral movement that manifests as squatting outward (Zhu et al., 2021), and during squatting, the knee joint produces a stretching effect from flexion to extension. Roberts, Lindstedt & Hoppeler (2016) showed that this stretching effect could increase the contraction force of the muscles and thus increase the muscle strength of the lower limbs (Wu & Hitt, 2005). Yan et al. (2022) and Jacobson et al. (1997) reported that the Tai Chi lateral movement step is a kind of exercise step with alternating feet that can increase the balance ability of the lower limb because the transition between the feet belongs to the process of power chain transformation. According to kinetic chain perspectives, the conversion of the force of one leg to the other during alternate foot movements in the ELS increases the force load, which in turn increases the force on the ground and can enhance limb control and balance (Lam et al., 2019; Reid, Elliott & Alderson, 2008), which in turn shows an increasing trend in ground reaction force. As reported in Matijevich’s study, increases in GRF metrics could not indicate increases in the overuse injury risk; in contrast, it is possibly related to muscle strength and contributions (Matijevich et al., 2019). Therefore, the increase in ground reaction force can effectively stimulate the joints and muscles of the lower limbs (Gao et al., 2020; Zhang et al., 2021). Therefore, long-term adherence to the lateral movements of Tai Chi (Bafa Wubu) can promote contraction training of the lower limb muscles.

### Lower extremity joint load analysis

Bafa Wubu of Tai Chi is a slow-to-fast and fast-to-slow sport where many motions are based on half squats (Chang et al., 2016). This article compared load at lower extremities during seven motions in Tai chi (Bafa Wubu) and discovered that hip joint load significantly increases both in the sagittal axis during SKR and in the vertical/frontal axis during WOF, LF, and RBB. These four movements result in increased forces in the hip joint, which Duan et al. (2019) attributed to a greater range of motion and improved hip coordination. Uhlig (2012) reported in a systematic review that this training modality also helps well in patients with osteoarthritis of the hip joint. Thus, the four movements of Bafa Wubu promote a range of motion in the hip joint. In terms of knee joint force, the knee joint force in the vertical direction was significantly increased in the seven sets of movements of Tai Chi (Bafa Wubu), which was related to the fact that all seven sets of movements of Bafa Wubu consisted of a bow stance. The bow stance is primarily a flexion and extension movement by the knee joint (Escalante et al., 2010; Li et al., 2019). Chang, Chang & Huang (2014) showed that bow stance training in Tai Chi requires more knee flexors and extensors to be involved in lower limb stabilization, which in turn increases the knee joint forces, and compared with other Tai Chi, Tai Chi (Bafa Wubu) has this unique step pattern movement for each movement, which can effectively enhance the flexion and extension strength of the knee joint. In terms of ankle joint force, ankle joint load increases significantly in the vertical axis during RBB, PB, and PPS. These three movements are characterized by backward movement, and backward movement is a relatively uncommon form of movement that requires a toe landing and then a heel landing, during which the ankle joint needs to perform flexion and extension movements to control the stability of the landing (Wu et al., 2019; Balasukumaran, Gottlieb & Springer, 2020). Thus, Tai Chi (Bafa Wubu) is a training method that can increase ankle joint stress and enhance ankle joint flexion and extension strength. Ankle flexion and extension strength is significant in daily life. Pol et al. (2022) study pointed out that insufficient ankle strength often leads to fall injuries during walking, while Bafa Wubu, a backward moving step training, can promote ankle strength and improve ankle support when landing during walking (Yang & Liu, 2020).

Additionally, joint torque is a key element of muscle activities around joints (Yu et al., 2021b), which plays a pivotal role in motion. This article discovered that hip flexion/extension/abduction/rotation torque significantly increases during RBB and PB, knee flexion/extension torque increases during RBB and ELS, and ankle flexion/extension torque increases during PB, which results from the five moving directions in Bafa Wubu exercise lower extremity joints in different ways. In Bafa Wubu, stepping backward and sideways effectively supplements original unidirectional movement, enriching the diversity of lower extremity exercise, which also helps improve the flexibility of the hip joint (Law & Li, 2014; Cheng et al., 2017) and improves the kinesthesia of the knee and ankle (Shaffer & Harrison, 2007; Hu, Lai & Wang, 2019).

### Lower extremity muscle activity analysis

The WOF and LF depicted in this article involve a slow landing from heel off to toe-off, which results in significantly longer and more frequent activation of the plantar flexor and dorsiflexors at the ankle (Li & Law, 2017), as well as greater stimulation of the muscles of the lower extremities (Wang, Xu & Li, 2016). In a report by Uhlig et al. (2010), it was shown that the slow lower limb step movements of Tai Chi require slow control of the ankle joint, which in turn requires increased flexor and extensor muscle strength. Therefore, the two forward direction movements of Tai Chi (Bafa Wubu) help to strengthen the plantar flexors and dorsiflexors. In this article, we find that WOF and LF are forward moving, but LF contains a force of external rotation in addition to forward force. When the hip joints generate a force forward, their external rotation is complemented simultaneously, which can be proven by the professional participants’ greater muscle strength of the vastus lateralis, vastus medialis, and gluteus minimus. This is because Bafa Wubu is characterized by rotation and twisting such that knee etensor and flexor muscles and hip abductors are responsible for stepping forward, whereas amateurs tend to ignore the added strength of rotation. In a study by Penn et al. (2019), it was concluded that this unique external rotation force of Tai Chi could promote the exercise of the lower extremity adductor muscles.

This article found that when professional participants step backward, hip abductors, hip adductors, and knee extensor and flexor muscles are responsible for providing strength. Since both RPB and PB require force backward, downward, and outward with the center of gravity shifting between the lower extremities, the joints of the hip and knees are extended and flexed, while the lower extremity muscles undergo repeated concentric and eccentric contractions (Hill et al., 2020). Hence, Bafa Wubu is of great value to increase the muscle strength of the lower extremities (Cui et al., 2017). This study confirms that long-term training of Tai Chi (Bafa Wubu) backward movements can promote the exercise of hip abduction muscles, hip adduction muscles, and knee extension and flexion muscles.

In addition, Bafa Wubu of Tai Chi contains special motions in the med-lat direction. This study found that the hip abductors and hip adductors of the professional participants, including the adductor longus, vastus lateralis, vastus medialis, sartorius, and gluteus minimus, displayed significantly more strength than amateur participants. This proves that lateral movements effectively exercise muscles on both sides of the thigh and strengthen the hip abductors and hip adductors, which activates the lateral nerves and muscles and improves the lateral balance capacity (Chen et al., 2021). Through the research of Zhang et al. (2022) and Sinsurin, Valldecabres & Richards (2020), the level of active and passive muscles on both sides of the leg can reflect the stability of the lower limbs. People lack training of the muscles on both sides of the thigh during daily walking and other exercise movements. In contrast, the lateral movements of Tai Chi (Bafa Wubu) can promote muscle strength training on both sides of the leg. During SKR, participants must stand unassisted on one leg with another knee raised and remain balanced. The professional participants’ iliacus of the raised leg exerted the most force during SKR, which may result from the height of the raised leg. The iliacus, controlling the abduction and rotation of the hip joints, plays an essential role in balancing body postures (Alam et al., 2019), so it is responsible for single leg balance. For amateurs, SKR training strengthens the iliacus and efficiently improves their physical balance.

Strengths and limitations of the study: The advantage of this research is that the lower limb movement of the new Tai Chi (Bafa Wubu) is analyzed for the first time by computer modeling for the movement biomechanical characteristics. It scientifically reveals the movement characteristics and exercise effects of Tai Chi (Bafa Wubu), and provides scientific theoretical guidance for amateurs’ training.

This study provides a comprehensive analysis of the new Tai Chi (Bafa Wubu) exercise lower limb joints and muscles, but there are certain limitations in this article, mainly related to the sample size, which is small because there are special populations in the sample and national athletes were selected. In addition, only males were selected for this study, and female practitioners need to be included in the study to increase the sample size as much as possible to conduct a more comprehensive study.

## Conclusions

The research in this article shows that from practitioners who have been practicing Tai Chi (Bafa Wubu) for a long time, the long-term practice of Tai Chi (Bafa Wubu) can enhance the muscle strength of the lower limbs, as well as promote the control of the joints of the lower limbs, and play a role in promoting lower limb exercise.

Amateurs are likely to encounter problems in Bafa Wubu training, and the capacity to control their hip abduction and rotation is inadequate. Hence, practitioners are encouraged to appropriately strengthen their control of their hip joints. The iliacus should be strengthened for standing knee raises becauseit plays an essential role in stabilizing the lower extremity balance. In brief, to achieve optimal effects, practitioners are encouraged to pursue normative and continuous technical training of Bafa Wubu.

In addition, because of the small sample size, this study is mainly a preliminary study of Tai Chi (Bafa Wubu), which reveals the basic movement patterns of the new Tai Chi (Bafa Wubu) and lays the foundation for future research.

## Abbreviations

BMI: Body mass index
WOF: Warding off with steps forward
LF: Laying with steps forward
RBB: Rolling back with steps backward
PB: Plucking with steps backward
PPS: Pushing and pressing with steps sideways
ELS: Elbowing and leaning with steps sideways
SKR: Standing knee raises
flex/ext: flexion/extension

## Additional Information and Declarations

### Data Availability

The following information was supplied regarding data availability: The data is available at Figshare: Li, Haojie (2022): data.zip. figshare. Dataset. https://doi.org/10.6084/m9.figshare.21571602.v4.
